# Nonlinear Mixed-Effects Modeling to Characterize the Pharmacokinetics of a Novel Mithramycin Analogue for Ewing Sarcoma in Mice

**DOI:** 10.21203/rs.3.rs-9035594/v1

**Published:** 2026-04-02

**Authors:** Kumar Kulldeep Niloy, Jamie Horn, Nazmul Hasan Bhuiyan, Khaled A Shaaban, Suhas S Bhosale, Thomas Prisinzano, Jon S Thorson, Jurgen Rohr, Markos Leggas

**Affiliations:** St. Jude Children’s Research Hospital; St. Jude Children’s Research Hospital; University of Kentucky; University of Kentucky; University of Kentucky; University of Kentucky; University of Kentucky; University of Kentucky; St. Jude Children’s Research Hospital

**Keywords:** Population pharmacokinetics, Preclinical pharmacokinetics, Ewing sarcoma, mithramycin analogues

## Abstract

**Purpose:**

To develop a pharmacokinetic model for a novel mithramycin analogue, MTMSA-Trp, in mice and characterize dose-dependent disposition to support future pharmacokinetic-pharmacodynamic (PK/PD) and exposure-efficacy analyses.

**Methods:**

Non-linear mixed-effects modeling was used to develop a population pharmacokinetic (popPK) model in MonolixSuite 2024R1 using 121 plasma concentrations from 70 female athymic nude mice after single IV bolus doses of 0.3, 1, 3, 5, and 10 mg/kg. Model selection was guided by the objective function value (OFV), parameter precision, and diagnostic plots. The final model was evaluated using bootstrap resampling (1000 replicates) and visual predictive checks (VPC; 1000 simulated datasets).

**Results:**

A one-compartment model with first-order elimination and an empirical power relationship between dose and clearance best described the data. Including dose as a covariate in the clearance model significantly improved model fit relative to the linear base model (ΔOFV = – 26.19). Typical clearance and volume of distribution were 39.18 mL/h/kg (at 3 mg/kg) and 53.06 mL/kg, respectively, and the dose-clearance exponent was β = −0.30, indicating decreasing clearance with increasing dose. Fixed-effect parameters were estimated with high precision (RSE ≤ 11%). Shrinkage was high for clearance (81%) and moderate for volume of distribution (39.1%). Bootstrap and VPC results supported model robustness and predictive performance.

**Conclusion:**

A robust popPK model describing dose-dependent MTMSA-Trp disposition in mice was developed and is suitable for simulation to support subsequent PK/PD and exposure-efficacy analyses.

## Introduction

MTMSA-Trp is a newly synthesized analogue of mithramycin (MTM) with potent inhibition of EWS-FLI1 activity and strong antitumor effects in Ewing sarcoma mouse xenograft models [1–4]. In addition to improved target engagement and in vivo efficacy, MTMSA-Trp has demonstrated improved pharmacokinetics compared with MTM across multiple species [5].

A key step in the preclinical development of any new compound is characterizing its PK in relevant species. MTMSA-Trp pharmacokinetics have been evaluated in female athymic nude mice, a commonly used xenograft host, following single IV bolus doses of 0.3, 1, 3, 5, and 10 mg/kg. These studies used mixed sampling approaches (serial and terminal/destructive sampling), resulting in sparse data. Noncompartmental analysis (NCA) provided clearance estimates ranging from 33 to 69 mL/h/kg and suggested non-proportional exposure across the dose range[5].

While NCA is useful for summarizing pharmacokinetics, population PK modeling provides a flexible framework for integrating pooled data across studies, quantifying inter-individual variability, and supporting simulation, especially when sampling is sparse [6, 7]. A validated popPK model enables prediction of exposures under alternative dosing regimens and provides the quantitative foundation for PK/PD and exposure-response modeling.

Accordingly, the objective of this study was to develop a popPK model for MTMSA-Trp in female athymic nude mice using pooled plasma concentration-time data from multiple studies and to evaluate dose dependence in pharmacokinetics across the dose range of 0.3 to 10 mg/kg [5]. The resulting model is intended to provide robust population parameter estimates as inputs in simulations to support the preclinical development of MTMSA-Trp for Ewing sarcoma.

## Materials and Methods

### Mice

All mice were handled in accordance with the Guide for the Care and Use of Laboratory Animals (National Research Council, 2011) standards. Studies were performed at St. Jude Children’s Research Hospital under an institutionally approved protocol (St. Jude Institutional Animal Care and Use Committee protocol 3164, approved 29 March 2022). Additionally, mouse experiments were performed at the University of Kentucky under institutionally approved protocols (University of Kentucky Institutional Animal Care and Use Committee protocols 2016–2345, approved 14 February 2019, and 2018–2849, approved 15 June 2021). Athymic nu/nu mice were purchased from Envigo RMS (Indianapolis, IN, USA) and housed in AAALAC-certified, temperature- and humidity-controlled (64°F to 84°F and 30% to 70%, respectively) facilities with a 12 h light-dark cycle. Mice were provided standard commercial diets (rodent 5R53 Test Diet, Richmond, IN, USA) and chlorinated, reverse-osmosis-treated water ad libitum. Animals were allowed to acclimate for at least a week prior to experimentation.

#### Pharmacokinetic data

Plasma concentration-time data were obtained from pharmacokinetic studies conducted in female athymic nude mice. MTMSA-Trp was administered as a single intravenous (IV) bolus dose of 0.3, 1, 3, 5, or 10 mg/kg [5]. Sampling followed both serial and terminal/destructive designs, resulting in a sparse dataset in which some mice contributed a single observation. MTMSA-Trp plasma concentrations were quantified using a validated LC-MS/MS bioanalytical method [8].

#### Software

Population pharmacokinetic analyses were conducted using MonolixSuite 2024R1 (Simulations Plus) with the stochastic approximation expectation–maximization (SAEM) algorithm for parameter estimation. Model-based simulations were performed in Simulx 2024R1 (Simulations Plus). Diagnostic and presentation plots were generated using GraphPad Prism 10.

#### Model development

One- and two-compartment structural models were evaluated to describe MTMSA-Trp plasma concentration-time data across the studied dose range. Models with first-order elimination were evaluated first. A Michaelis-Menten elimination model was also explored but did not yield stable parameter estimates and was not retained.

Between-subject (inter-individual) variability (IIV) in pharmacokinetic parameters was modeled using an exponential (log-normal) model:

[Equation 1] $$P_{i}=P_{\text{pop}}.e^{\eta_{i}}$$

where *P*_*i*_ is the individual value of parameter *P* for animal *i*, and *P*_*pop*_ is the typical population value for parameter *P*. Depending on the structural model, *P* included clearance (CL) and volume of distribution (V), and for two-compartment models also included intercompartmental clearance (Q) and peripheral volume (*V*_*p*_). The random effect *η*_*i*_ describes the deviation of animal *i* from the population typical value and was assumed to be normally distributed with mean 0 and variance *ω*^2^ (that is, *η*_*i*_~N(0,*ω*^2^)). Additive, proportional, and combined residual error models were evaluated to describe residual unexplained variability.

Following selection of the base structural model, dose was tested as a covariate on apparent clearance using a power function to capture non-proportional exposure across dose levels:

[Equation 2] $${CL}_{i}={CL}_{\text{pop}}.{\left(\frac{{\text{Does}}_{i}}{{\text{Does}}_{\text{median}}}\right)}^{\beta }.e^{\eta_{CL,i}}$$

where *CL*_*pop*_ is the typical population clearance, *Dose*_*i*_ is the dose administered to animal *i*, *Dose*_*median*_ was set to 3 mg/kg, and *β* is the exponent estimating the strength of the dose effect.

Model selection and covariate retention were guided by objective function value (OFV; −2 loglikelihood) and parameter precision. For nested models, a decrease in OFV of ≥ 3.84 (p < 0.05; 1 degree of freedom) was considered statistically significant. Precision was assessed using relative standard error (RSE), with RSE < 30% for -fixed effect parameters and < 50% for -random effect- parameters were considered acceptable [9]. Shrinkage was also assessed to gauge the reliability of empirical Bayes estimates (EBEs), recognizing that sparse sampling can increase shrinkage and reduce the interpretability of EBE -based diagnostics [10, 9, 11].

#### Model evaluation

Model adequacy was evaluated using standard graphical and simulation-based diagnostics. Observed concentrations were compared with population predictions (PPRED) and individual predictions (IPRED). Population-weighted residuals (PWRES), individual-weighted residuals (IWRES), and normalized prediction distribution errors (NPDE) were examined as functions of time and predictions to assess bias and model misspecification.

Parameter stability and precision were assessed using non-parametric bootstrap resampling (1000 replicates); bootstrap medians and 95% confidence intervals were compared with final parameter estimates. Predictive performance was evaluated using visual predictive checks (VPCs) based on 1000 simulated datasets generated under the original study design (dose levels, group sizes, and sampling times), with observations overlaid on the simulated 5th, 50th, and 95th percentiles.

To evaluate the dose effect on clearance, dose-stratified VPCs were generated and compared between the base (linear) and final (dose-dependent) models. As an additional verification of the dose-exposure relationship, concentration-time profiles of 1000 virtual mice were simulated for each dose group (0.3, 1, 3, 5, and 10 mg/kg) using the final model. Noncompartmental analysis was performed on the simulated data to calculate AUC_0àinf_ and dose-normalized AUC_0àinf_, which were plotted versus dose.

## Results

### Pharmacokinetic data

**Figure 1:**
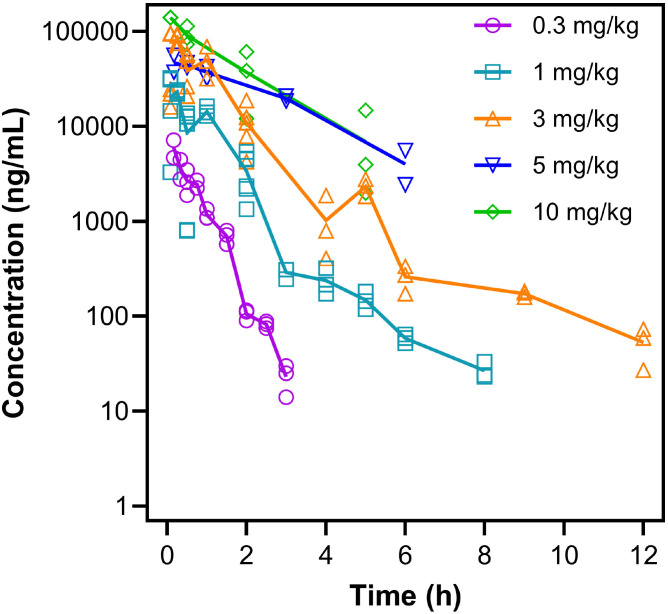
Observed MTMSA-Trp plasma concentrations (symbols) over time across dose range used for model development. Solid lines represent mean PK profiles.

The popPK dataset comprised 121 plasma concentrations from 70 mice collected following single IV bolus administration of MTMSA-Trp at doses ranging from 0.3 to 10 mg/kg [5]. Data were pooled across studies that used both serial and terminal/destructive sampling, resulting in a sparse design in which terminal animals contributed a single concentration. A summary of the analysis dataset is provided in **Table S1**, and observed concentration-time profiles across dose groups are shown in Fig. 1.

#### Model development

One and two-compartment models with first-order elimination were evaluated, along with additive, proportional, and combined residual error models. A Michaelis-Menten elimination model was explored but produced unstable parameter estimates and was not retained. A one-compartment model with first-order elimination, lognormal IIV on CL and V, and a proportional residual error model adequately described the data and was selected as the base model. The two-compartment model did not improve fit despite added complexity (**Table S2**).

Dose was then evaluated as a covariate on CL using a power model. Inclusion of the dose effect significantly improved model fit relative to the base model (ΔOFV = – 26.19; **Table S2**) and was retained in the final model.

Final parameter estimates are summarized in Table 1. Typical population CL and V were 39.18 mL/h/kg and 53.06 mL/kg, respectively, and the dose-CL exponent was *β*=−0.30, indicating decreasing apparent clearance with increasing dose. Fixed effect- parameters were estimated with good precision (RSE ≤ 11%). Incorporation of the dose effect reduced unexplained IIV on CL (15% to 5.8%) and decreased IIV on V (58% to 25%; **Table S3**). Shrinkage was high for CL (81%) and moderate for V (39.1%), consistent with the sparse/destructive sampling design.

### Model evaluation

**Figure 2:**
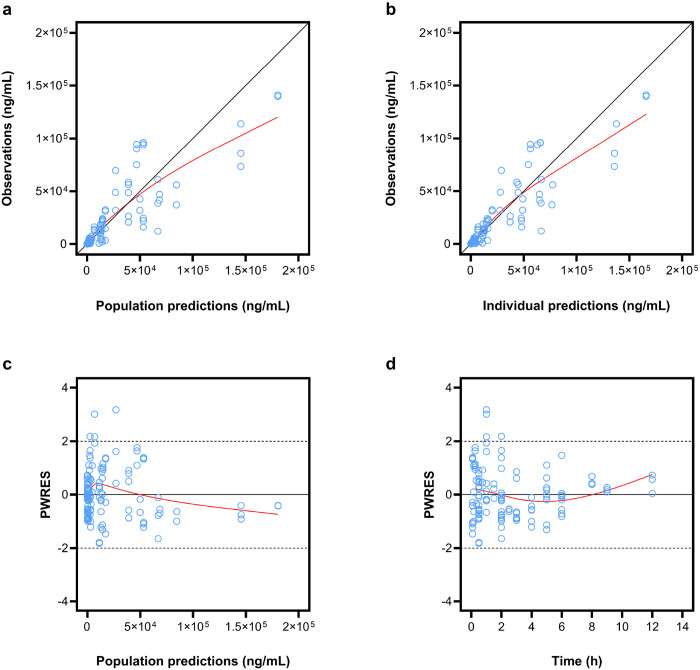
Goodness-of-fit plots of final popPK model. Panel a and b show the observations versus population (PPRED) and individual predictions (IPRED), respectively. Panel c and d show population weighted residuals (PWRES) versus population predictions (PPRED) and time, respectively. Black and red lines represent the line of unity and spline, respectively.

Goodness-of-fit diagnostics demonstrated acceptable agreement between observed concentrations and model predictions (Fig. 2). Observations were symmetrically distributed around the line of identity in plots of observed versus PPRED and IPRED, and PWRES/IWRES/NPDE were centered around zero without systematic trends versus time or predictions (Fig. 2; **Supplementary Fig. S1**). Given the sparse sampling design and associated shrinkage for clearance, EBE-based diagnostics were interpreted cautiously, and emphasis was placed on simulation-based evaluation.

**Figure 3:**
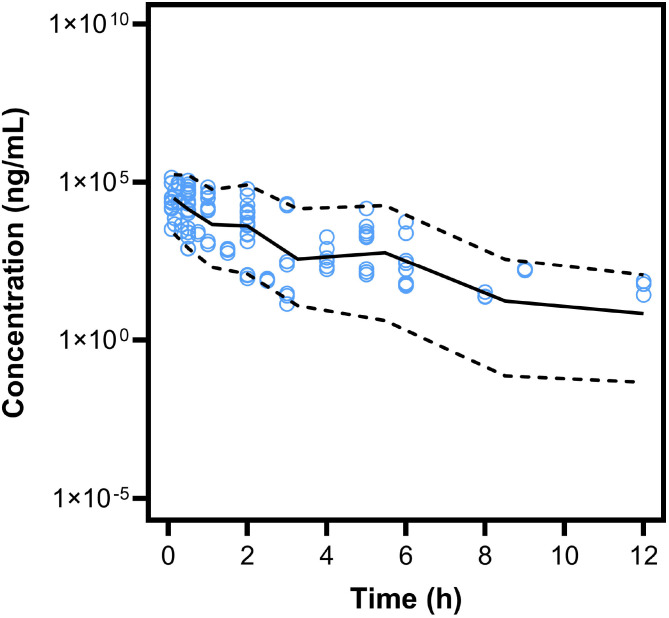
Visual predictive check of final popPK model following 1000 simulations. Solid line represents median and dashed lines represent 5th and 95th percentiles. Blue circles represent observations.

**Figure 4:**
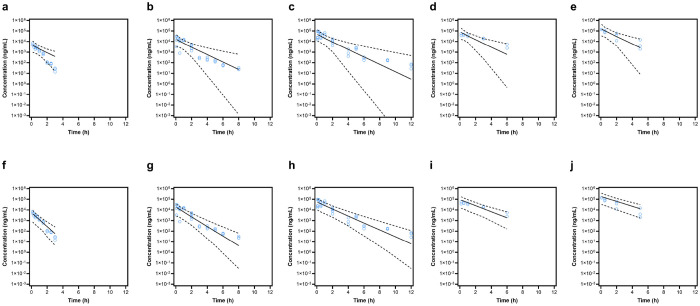
Comparison of VPC plots between base and final popPK models. Top panel (a-e) and bottom panel (f-j) show VPCs for base and final models, respectively, for 0.3 mg/kg (a & f), 1 mg/kg (b & g), 3 mg/kg (c & h), 5 mg/kg (d & i) and 10 mg/kg (e & j).

Overall, VPC results indicated that the majority of observed concentrations fell within the simulated 5th-95th prediction interval and were well described by the simulated median (Fig. 3), supporting predictive performance for simulation. Comparison of overall and dose-stratified VPCs showed that the base linear model did not adequately capture the profile at 0.3 mg/kg and did not fully account for variability at later time points, whereas the final model with dose-dependent clearance improved agreement across doses (Fig. 4; **Supplementary Fig. S2**).

**Figure 5:**
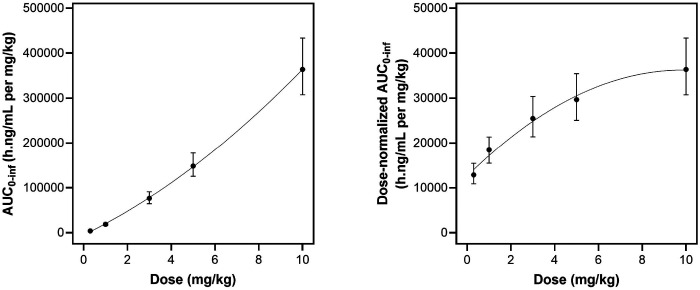
Dose dependent nonlinearity plots. Panel a shows exposure versus dose and panel b shows dose-normalized exposure versus dose. The black dots represent median simulated exposures and error bars represent upper/lower limits.

Simulation-based evaluation of exposure supported the estimated dose effect on clearance. Simulated AUC_0àinf_ increased more than proportionally with dose, and simulated dose-normalized AUC_0àinf_ increased with dose (Fig. 5), consistent with β < 0 and decreasing clearance with increasing dose over the 0.3 to 10 mg/kg range.

Bootstrap resampling (1000 replicates) confirmed model stability. Final parameter estimates were consistent with bootstrap medians and lay within the bootstrap 95% confidence intervals (Table 1), supporting the robustness and precision of the final model.

## Discussion

Using sparse plasma concentration-time data pooled across studies in female athymic nude mice, we developed a popPK model to characterize MTMSA-Trp disposition over a wide dose range (0.3 to 10 mg/kg). The primary goal was to obtain robust population parameter estimates suitable for simulation efforts to support future PK/PD and exposure-response analyses.

A one-compartment model with first-order elimination provided a parsimonious description of the data and was preferred over a two-compartment model, which did not improve the fit. However, diagnostics from the linear base model suggested dose-dependent behavior, most notably at the lowest dose and in the extent of variability at later sampling times. Incorporating dose as a covariate on clearance using an empirical power model substantially improved fit (ΔOFV = – 26.19) and improved predictive performance in both overall and dose-stratified VPCs. Although a mechanistic Michaelis-Menten elimination model was explored, it did not yield stable parameter estimates; therefore, the retained power model should be interpreted as an empirical description of non-proportional exposure within the studied dose range rather than a definitive mechanism.

The final model captured the central tendency and variability across doses, as shown by the dose-stratified VPCs, and provides an empirical description of dose-dependent clearance within the therapeutically relevant range [1, 2, 12]. The final model estimated β = −0.30, indicating decreasing clearance with increasing dose and a greater-than-proportional increase in exposure (AUC_0àinf_) across 0.3 to 10 mg/kg. Typical clearance at the reference dose (3 mg/kg) was consistent with prior NCA estimates, supporting concordance with earlier analyses. The estimated volume of distribution (~ 53 mL/kg) is physiologically plausible and close to mouse plasma volume (~ 50 mL/kg) [13] suggesting distribution largely confined to the vascular space, consistent with prior observations of high plasma protein binding.

Incorporating dose as a covariate on CL reduced unexplained IIV in clearance (15% in the base model to 5.8% in the final model), indicating that a substantial portion of the apparent variability in clearance across animals was explained by dose within the studied range. An important limitation, however, was the high shrinkage estimated for CL (81%). High shrinkage is expected when individual information content is limited (e.g., sparse or destructive sampling) and indicates that EBEs for CL are pulled toward the population mean. Under these conditions, IPRED may closely resemble PPRED, and EBE-based diagnostics should be interpreted cautiously.

Accordingly, model evaluation emphasized simulation-based diagnostics (VPC and NPDE) and - population level parameter precision (bootstrap). The final model yielded precise population estimates (RSE ≤ 11%), bootstrap medians consistent with final estimates, and simulation-based diagnostics without systematic bias. Given the primary -objective to characterize the population PK of MTMSA-Trp from sparse data to support subsequent exposure-response analyses, the final model was considered suitable for its intended purpose. Importantly, the dose-CL relationship should be interpreted as an empirical description of non-proportional exposure over 0.3 to 10 mg/kg, rather than a definitive mechanistic identification of the source of nonlinearity.

In conclusion, a one-compartment popPK model incorporating dose-dependent clearance provided a stable and predictive description of MTMSA-Trp pharmacokinetics in mice across 0.3 to 10 mg/kg. The model yields robust population parameter estimates suitable for simulation and provides a quantitative framework for future PK/PD, exposure-efficacy, and translational modeling efforts.

## Supplementary Material

Supplementary Files

This is a list of supplementary files associated with this preprint. Click to download.

- supplementarymaterials.docx

## Figures and Tables

**Table 1:** Population PK model parameters.

Parameter	Estimate (%RSE)	Bootstrap (n = 1000)		Shrinkage (%)
		Median	95% CI	
*Fixed effect*				
CL (mL/h/kg)	39.18 (4.97)	37.88	31.78–45.76	-
V (mL/kg)	53.06 (7.55)	51.48	42.06–64.84	-
β	−0.30 (10.9)	−0.31	−0.40 – −0.18	-
*Interindividual variability*				
IIV on CL (CV%)	5.8 (42)	0.061	0.028–0.14	81
IIV on V (CV%)	25 (31)	0.21	0.11–0.40	39.1
*Residual variability*				
b (%)	0.51 (8.13)	0.5	0.41–0.58	-

## Data Availability

Inquiries regarding the datasets should be directed to the corresponding author.
